# A Standard Procedure for In Vitro Digestion Using Rumen Fermenters: A Collaborative Study

**DOI:** 10.3390/ani12202842

**Published:** 2022-10-19

**Authors:** Larissa Frota Camacho, Tadeu Eder da Silva, João Paulo Pacheco Rodrigues, Marcia de Oliveira Franco, Edenio Detmann

**Affiliations:** 1 Department of Animal Science, Universidade Federal de Viçosa, Viçosa 36570-900, MG, Brazil; 2 Department of Animal and Dairy Sciences, University of Wisconsin, 1675 Observatory Drive 266, Animal Sciences Building, Madison, WI 53706-1205, USA; 3 Department of Animal Production, Universidade Federal Rural do Rio de Janeiro, Seropédica 23897-000, RJ, Brazil; 4 Natural Resources Institute Finland (Luke), FI-31600 Jokioinen, Finland; 5 Department of Animal Nutrition and Management, Swedish University of Agricultural Sciences, 750 07 Uppsala, Sweden

**Keywords:** artificial fermenter, Horwitz ratio, in vitro digestibility, repeatability, reproducibility

## Abstract

**Simple Summary:**

Knowledge of the nutritive value of feeds is essential to feed animals with adequate diets and to optimize production with minimal environmental impact. In vitro digestibility might be an important source of information for nutritionists, because it is a cheap and fast way to assess information on feed digestion for ruminants, notably when the main objective is to compare feeds or diets. A rumen fermenter is a relatively new type of equipment which improves the operational capacity of in vitro procedures. However, the lack of standardized procedures for in vitro trials using rumen fermenters may compromise the reliability of information obtained, mainly due to the high variability among laboratories. Hence, we proposed and evaluated a standard method for in vitro digestion using rumen fermenters, through a collaborative study involving seven feed analysis laboratories. On average, the method showed adequate performance, where the random variation among laboratories was lower than the random variation within laboratories (i.e., error variation). Considering that in vitro digestibility is an analytical entity that is defined by the method itself, the proposed method was considered reproducible. Our results highlighted that, if the method is followed exactly, its results present adequate levels of repeatability and reproducibility.

**Abstract:**

Our objective was to propose and evaluate a standard procedure for the evaluation of in vitro dry matter digestibility for ruminant feeds, using artificial fermenters. A collaborative study was performed with seven feed analysis laboratories and four feeds (Tifton 85 hay, corn silage, soybean hulls, and soybean meal). Two types of artificial fermenters were evaluated (DaisyII Ankom and TE-150 Tecnal). Each laboratory received 80 sealed filter bags with samples (20 per feed), eight blank filter bags, a plastic bag with buffer solution reagents, and instructions describing how to conduct a 48 h in vitro assay using an artificial fermenter and how to collect bovine ruminal inoculum. On average, the contribution of laboratory effect to the total random variance was 24%, being less than the contribution of equipment (42%) and error (34%). The repeatability ranged from 3.34 to 5.79%, across feeds. The reproducibility ranged from 5.93 to 8.94% across feeds, which implied Horwitz ratios ranging from 2.94 to 4.10. Due to the specific characteristics of the analytical entity evaluated here, which is defined by the method itself, the proposed method was considered reproducible. The results highlighted that, if the method is followed exactly, its results are precise and present adequate levels of repeatability and reproducibility.

## 1. Introduction

Initially, in vitro digestibility assays were proposed in order to estimate forage in in vivo digestibility [1]. However, currently the range of application of these kind of techniques has increased, mainly for screening, discrimination, or direct comparison of feeds and diets [2]. This kind of technique is mostly used because it is fast, relatively inexpensive, and precise.

However, the apparent in vitro dry matter digestibility (or indigestibility) is an analytical entity defined by the method itself. Thus, they are methods that determine a value that can only be obtained in terms of the method itself [3]. As there are no primary reference standards for this type of method, they cannot be validated for accuracy in determining the “true” value of the constituent. To minimize systematic errors (bias) among laboratories, empirical methods must be followed exactly as described in the standard manuals. Even slight variations in the method may result in the measurement of a different constituent [4].

The in vitro digestibility can be affected by several alterations in the standard procedures, such as laboratory instruments, vessel and filter bag types, buffer solutions, headspace gas type, way of incubating samples, analyst working, inoculum sources, inoculum donor diet, sample grinding, and others [2,5,6,7,8,9,10,11]. Any change either in the number of steps or in any parameter of the analysis will result in different in vitro methods whose digestibility estimates cannot be directly compared with each other.

Indeed, the among-laboratory variation tends to be greater for empirical methods (i.e., type I methods), because analysts often perform these methods in nonstandard ways that do not follow the official method. In addition, quality assurance programs established to verify results in laboratories are often inadequate or even nonexistent. Sometimes, the limitations of methods and the background for specific steps in a method have not been published or have not been appropriately communicated to the analyst. Most of the among-laboratories variation is associated with the analysts’ desire to improve efficiency by shortening times, eliminating steps, or failing to follow the details of a method and assuming that those deviations could not be significant enough to affect the results. These sometimes well-intentioned deviations ignore the fundamental property of the empirical methods, which requires that they be followed exactly [4].

Compared to conventional methods, the utilization of artificial rumen fermenters and filter bags simplifies in vitro digestibility measurement, eliminating the need for filtering samples after digestion, which is often one of the most labour-intensive steps in the conventional procedure. The incubation of several samples within a jar also reduces the need for individual inoculation of samples in tubes [5]. Despite those aspects, a standard and widely applied method for evaluating in vitro digestibility of ruminant feeds and diets using rumen fermenters that produces reliable and comparable results, would allow minimizing the variability among laboratories and carrying out a more reliable comparison among feeds and diets offered to cattle and other domestic ruminants.

In order to do this, the study director’s laboratory at the Brazilian National Institute of Science and Technology in Animal Science (INCT-CA) developed several studies to establish standard methods of in vitro and in situ digestibility for ruminant feeds or diets (e.g., [2,10,12,13]). An integrated method using rumen fermenters was derived from those studies and has been successfully applied within the study director’s laboratory. In order to check the adequacy and reproducibility of that method, a collaborative study to estimate the in vitro dry matter digestibility for ruminant feeds was conducted, following the standard procedure proposed by the Brazilian National Institute of Science and Technology in Animal Science.

## 2. Materials and Methods

All animal care and handling procedures applied in this work were approved by Ethics Committee on the Use of Production Animals of the Universidade Federal de Viçosa (protocol number 029/2019).

### 2.1. Standardization of the Machine-Rinsing Procedure for Filter Bags

Prior to the collaborative study, a separate experiment was performed, aiming to define a standard machine-rinsing procedure for filter bags after incubation. The results obtained in this experiment were posteriorly incorporated into the in vitro digestibility method. The theoretical background for this experiment will be adequately presented in Section 3. The experiment was carried out at the Animal Nutrition Laboratory (the Study Director’s laboratory) of the Animal Science Department of the Universidade Federal de Viçosa, Viçosa, Minas Gerais, Brazil.

Four feed samples were used: Tifton 85 hay (*Cynodon* sp.), corn silage (*Zea mays*), soybean meal, and soybean hulls. These feeds were chosen to create a small but representative group of feeds, used to feed ruminants in the tropics. Corn silage sample was oven-dried (55 °C) and, along with the other feeds, was processed in a knife mill to pass through a 1-mm screen sieve. The samples were quantified regarding dry matter (DM) content (oven-drying at 105 °C for 16 h, method G-003/1; [14]).

The in vitro digestibility assay was performed in an artificial fermenter (TE-150, Tecnal Equipamentos Científicos, Piracicaba, São Paulo, Brazil; [2]). Twenty 500 mg test portions per feed were weighed and stored in heat-sealed filter bags (non-woven textile 100 g/m^2^; 4 × 4.5 cm; [12]). A rumen-cannulated bull, fed a sugarcane- and concentrate- (220 g of crude protein/kg DM) based diet with a forage-to-concentrate ratio of 80:20, was used as the inoculum donor. The animal had free access to water and a mineral mixture (90 g/kg of phosphorus), and was adapted to the diet for 14 days prior to rumen inoculum collection [15]. The ruminal inoculum (liquid and solid digesta) was collected at several points in the rumen, shortly before the beginning of incubation. Ruminal inoculum was stored in preheated (39 °C) thermal bottles and then mixed for a few seconds, using a blender (NL-26,400 W, Mondial, Conceição do Jacuípe, Bahia, Brazil), to homogenize liquid and solid phases. The fluid was then filtered through four layers of cheesecloth. The steps from rumen inoculum collection to incubation onset were conducted within 20 min in a climate-controlled room (39 °C). The artificial fermenter possessed four jars (3200 mL), and each jar randomly received all test portions of each feed and two blank bags. In each jar, 400 mL of ruminal inoculum and 1600 mL of McDougall’s buffer solution were added. The preparation of buffer solution followed the procedures described by Camacho et al. [10]. Carbon dioxide was flushed into the headspace of each jar, which was closed and placed into the preheated (39 °C) artificial fermenter. After 48 h of incubation, the filter bags were superficially washed with distilled water and gently pressed to remove gases.

All bags were placed in a washing machine (Turbilhão 5 kg model, Suggar, Belo Horizonte, Minas Gerais, Brazil). The machine was filled with clean tap water and a rinse cycle of 1 min of agitation (delicate setting) was used [16]. After that, the residual water was drained and bags were gently pressed to remove excess of liquid, oven-dried (55 °C/24 h and 105 °C/16 h, sequentially), placed in a desiccator, and weighed. This rinsing procedure was repeated seven times with all filter bags.

The apparently undigested DM residue was estimated as follows:

(1) $$UR=\frac{R-B}{M}\times 100$$

where *UR* is the apparently undigested residue (% DM), *M* is the incubated mass of DM (g), *R* is the undigested residue inside the bag (g), and *B* is the residual DM in blank filter bags (g).

The UR was submitted to an analysis of variance, including the fixed effects of feeds and rinsing and their interaction. The sequential rinses were considered as repeated measures. The (co)variance residual matrix was modeled according to a heterogenous compound symmetry structure. This choice was based on the Akaike information criterion with correction. The least-square estimates of UR were compared in terms of differences between sequential rinses using the Tukey-Kramer approach, according to the following hypotheses:

(2) $$H_{0}:\mu_{i}-\mu_{i+1}=0$$

(3) $$H_{a}:\mu_{i}-\mu_{i+1}\neq 0$$

where *i* denotes the rinse number.

Degrees of freedom were estimated using the Kenward-Roger approach. Statistical analysis was performed using the GLIMMIX procedure of SAS. Significance was declared at *p* < 0.05.

### 2.2. Collaborative Study

The collaborative study was performed in seven feed-analysis laboratories in Brazil: the Study Director’s laboratory; Universidade Federal Rural da Amazônia, Parauapebas, Pará; Universidade Estadual Paulista Júlio de Mesquita Filho, Jaboticabal, São Paulo; Veterinary Medicine College, Universidade Federal de Minas Gerais, Belo Horizonte, Minas Gerais; Animal Science and Veterinary College, Universidade Federal da Bahia, Salvador, Bahia; Universidade Federal de Lavras, Lavras, Minas Gerais; and Agricultural and Environmental Sciences Institute, Universidade Federal de Mato Grosso, Sinop, Mato Grosso.

The laboratories were chosen based on the following criteria: 1. They must be associated with the Brazilian National Institute of Science and Technology in Animal Science 2. The following items should be available in the laboratory: rumen-cannulated bovines, CO_2_ cylinder, and either a DaisyII (ANKOM Technology Co., Macedon, NY, USA) or a TE-150 (Tecnal Equipamentos Científicos, Piracicaba, SP, Brazil) artificial fermenter.

The feeds used in the previous experiment were also used as the study materials for the collaborative study. The DM (dried overnight at 105 °C, method G-003/1), crude protein (Kjeldahl procedure, method N-001/2), and neutral detergent fibre (NDF; method F-013/1) contents were analyzed in the Director’s laboratory of the Brazilian National Institute of Science and Technology in Animal Science, according to its standard analytical procedures [14] (Table 1). In particular, the NDF analysis was performed using a heat-stable α-amylase (Liquozyme Supra 2.2X, Novozymes, Araucária, Paraná, Brazil), omitting sodium sulphite, and expressed inclusive of residual ash and protein.

For the in vitro assay, test portions of 500 mg of each feed were weighed and stored in heat-sealed filter bags (non-woven textile 100 g/m^2^; 4 × 4.5 cm; [12]). Moreover, all reagents necessary to compose 10 L of McDougall’s buffer solution [10] were weighed and stored in labelled plastic bags.

Each laboratory received 80 sealed filter bags with test portions (20 per feed), eight blank filter bags, a plastic bag with buffer solution reagents, and instructions describing how to conduct a 48 h in vitro assay using an artificial fermenter and how to collect bovine ruminal inoculum. The complete method is fully described in the Supplementary Material. Briefly, as both types of artificial fermenters possess four jars each, the laboratories were instructed to use one jar for each feed (including two blanks per jar). After in vitro incubation, the laboratories superficially washed the filter bags with distilled water, and gently pressed them to remove gases. The bags were then oven-dried (55 °C/48 h) and sent back to the Study Director’s laboratory to estimate in vitro dry matter digestibility (IVDMD).

The filter bag rinsing procedure was performed in the Study’s Director laboratory, as in the previous experiment. The bags were placed in a washing machine (Turbilhão 5 kg model, Suggar, Belo Horizonte, Minas Gerais, Brazil). The machine was filled with clean tap water and a rinse cycle of 1 min of agitation (delicate setting) was set. The residual water was then drained. This procedure was repeated three times. After this, the bags were gently pressed to remove excess of liquid, oven-dried (55 °C/24 h and 105 °C/16 h, sequentially), placed in a desiccator, and weighed.

The apparent *IVDMD* was estimated as follows:

(4) $$IVDMD=\frac{M-\left(U-B\right)}{M}\times 100$$

where *IVDMD* is the in vitro dry matter digestibility (% DM), *M* is the incubated mass of DM (g), *U* is the undigested residue in the bag (g), and *B* is the residual DM in the blank filter bags (g).

The initial basic statistical model used to analyze *IVDMD* was:

(5) $$Y_{ijkl}=\mu +F_{i}+E_{j}+L_{\left(j\right)k}+\varepsilon_{ijkl}$$

where *Y_ijkl_* is the *IVDMD* of the test portion *l* of feed *i*, measured in the laboratory *k*, using the equipment *j*; *μ* is the general constant (fixed effect); *F_i_* is the random effect of feed *i*, assumed NIID (0, σ^2^_F_); *E_j_* is the random effect of equipment type *j* (i.e., artificial fermenter), assumed NIID (0, σ^2^_E_); *L_(j)k_* is the random effect of laboratory *k* nested within the equipment *j*, assumed NIID (0, σ^2^_L/E_); and *ε_ijkl_* is the random error, assumed NIID (0, σ^2^_ε_).

Despite the equipment effect being only two levels (i.e., DaisyII or TE-150), we decided to keep it as a random effect, as many other artificial fermenter brands are available on the market. Additionally, the laboratory effect was considered to be a nested effect of the equipment, in order to estimate the differences among laboratories without any further bias caused by using different artificial fermenters.

**Figure 1:**
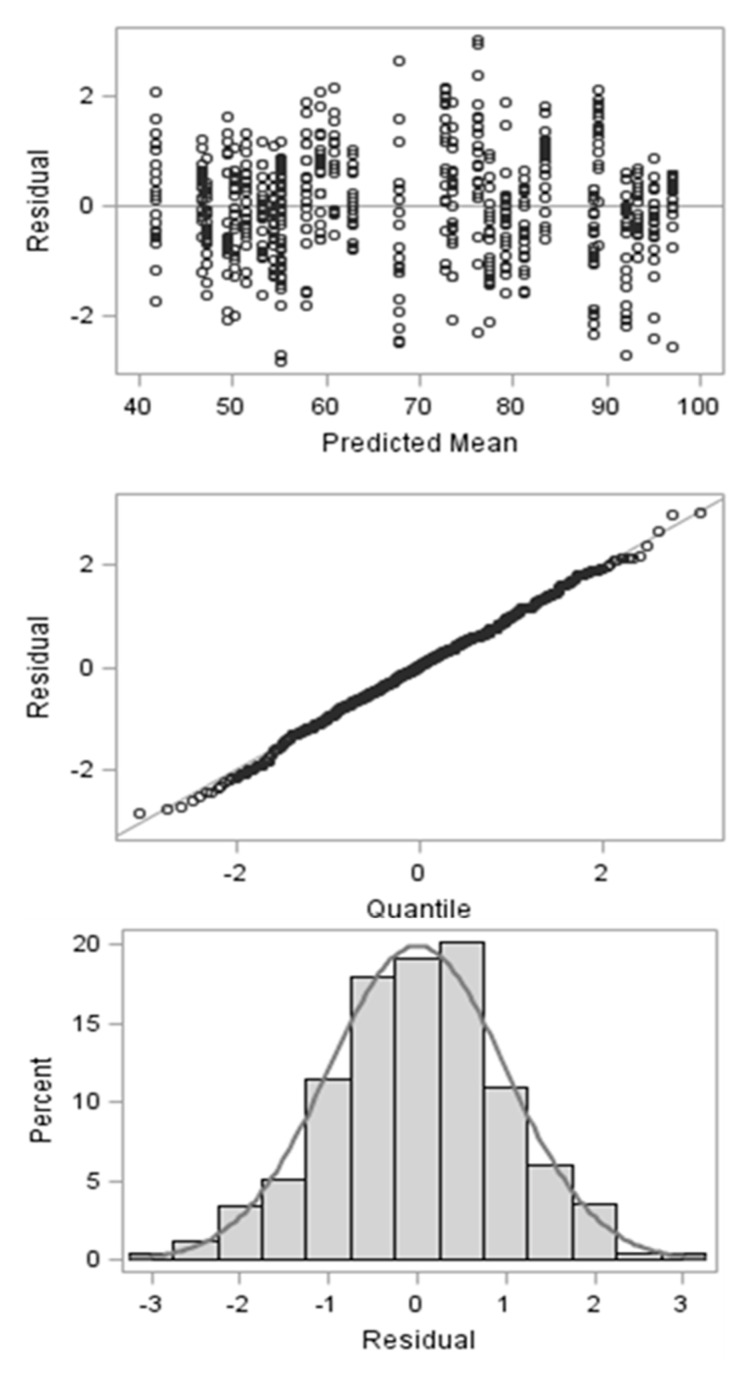
Descriptive pattern of studentized residues for in vitro dry matter digestibility after residual evaluation and outlier elimination.

Initially, we performed an outlier evaluation on the overall dataset. Three different criteria were defined in order to identify outliers: 1. Restricted likelihood distance > 0.3, COVRATIO < 0.8, and externally studentized residue (module) >2.5. An observation was considered as an outlier if it met at least two of those criteria. After this, only four observations were eliminated from the dataset (Table 2). The residues showed a clear pattern, agreeing with the assumption of a normal and homoscedastic distribution (Figure 1).

In order to improve the understanding on the pattern of the results, the IVDMD was also evaluated for each individual feed, according to the model

(6) $$Y_{ijk}=\mu +E_{i}+L_{\left(i\right)j}+\varepsilon_{ijk}$$

where *Y_ijk_* is the IVDMD of the test portion *k*, measured in the laboratory *j*, using the equipment *i*; *μ* is the general constant (fixed effect); *E_i_* is the random effect of equipment type *i* (i.e., artificial fermenter), assumed to be NIID (0, σ^2^_E_); *L_(i)j_* is the random effect of laboratory *j* nested within the equipment *i*, assumed to be NIID (0, σ^2^_L/E_); and *ε_ijk_* is the random error, assumed to be NIID (0, σ^2^_ε_).

From the adjustment of the models (5) and (6), the following technical performance indicators of the method were estimated [4,17,18,19]:

(7) $$S_{r}=\sqrt{{\hat{\sigma }}_{\varepsilon }^{2}}$$

(8) $$r=\frac{S_{r}}{\bar{Y}}\times 100$$

(9) $$S_{R}=\sqrt{{\hat{\sigma }}_{L/E}^{2}+{\hat{\sigma }}_{\varepsilon }^{2}}$$

(10) $$R=\frac{S_{R}}{\bar{Y}}\times 100$$

(11) $$S_{Re}=2\times C^{0.85}$$

(12) $$Re=2\times C^{-0.15}$$

(13) $$\mathrm{HotRat}=\frac{R}{Re}$$

where *s_r_* is the standard deviation of repeatability (intra-laboratorial variability), *r* is the repeatability (%), ${\hat{\sigma }}_{\varepsilon }^{2}$ is the estimate of error variance, $\bar{Y}$ is the average IVDMD (% DM), *s_R_* is the standard deviation of reproducibility (inter-laboratorial variability), *R* is the reproducibility (%), ${\hat{\sigma }}_{L/E}^{2}$ is the estimate of the variance among laboratories, *s_Re_* is the expected standard deviation of reproducibility, *Re* is the expected reproducibility (%), *C* is the average IVDMD (g/g DM), and *HorRat* is the Horwitz ratio.

Moreover, an adapted value of the Z-score [9] was calculated for each level of the random effects within each feed, according to the equation

(14) $$Z=\frac{\mathrm{eBLUP}}{SEp}$$

where *Z* is the adapted *Z*-score for the respective level of random effect (dimensionless), *eBLUP* is the empirical best linear unbiased predictor of the respective level of random effect, and *SEp* is the standard error of prediction associated with the *eBLUP*.

The laboratory eBLUPs were also used for applying a ranking laboratory performance test by adapting the protocols described by Wernimont and Spendley [20]. It must be noted that the test was applied using eBLUPs, rather than average IVDMD, as the former is adjusted for the effect of different equipment, which could bias the rank of laboratories within different feeds.

All statistical evaluations were performed using the MIXED procedure of SAS 9.4. The components of variance were estimated according to the restricted maximum likelihood method. When pertinent, significant results were declared at *p* < 0.05.

## 3. Results and Discussion

### 3.1. Standardization of the Machine-Rinsing Procedure for Filter Bags

One of the steps of the in vitro assays which is more dependent on analyst work is the rinsing procedures of filter bags. Practical recommendations sometimes rely on hand-rinsing procedures, the endpoint of which is subjectively defined by the water clarity [21]. In this sense, replacing hand-rising with a standard machine-rising procedure may reduce both the subjectivity of this method step and the variability among and within analysts. Despite the fact that some standardizations have been suggested for in situ procedures [16,22], a machine-rising procedure for filter bags used in in vitro assays has not yet been adequately defined.

**Figure 2:**
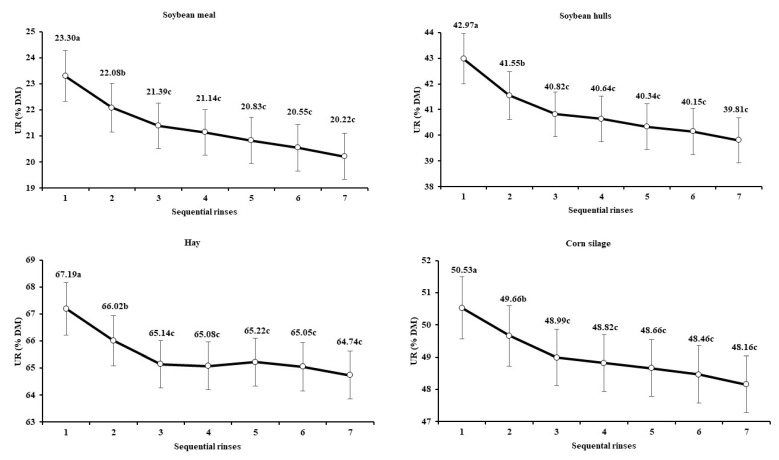
Least square means (±standard errors) for the apparently undigested residue (UR, % dry matter) in the different feeds and according to the number of rinses after in vitro incubation (means followed by different letters differ from sequential values at *p* < 0.05).

**Figure 3:**
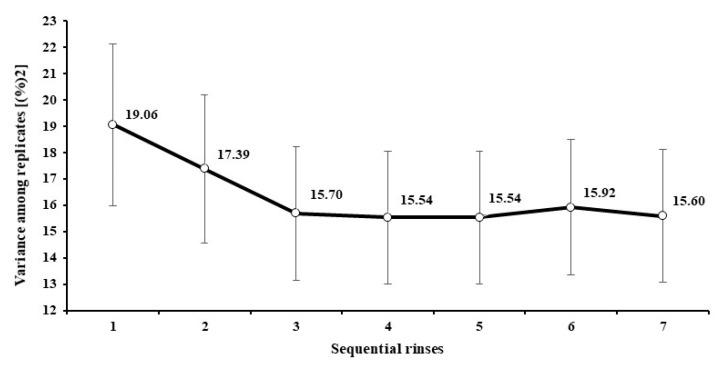
Estimates of variances among replicates (±standard error) for the apparently undigested residue, according to the number of rinses after in vitro incubation.

The analysis of variance indicated an interaction between feeds and number of rinses (*p* < 0.01). However, despite the interaction effect, all evaluated feeds showed the same pattern, as there was no significant change (*p* > 0.05) in UR after three rinses (Figure 2). In addition to the UR decrease as the number of rinses increased, the variance among replicates also decreased and was minimized from the third rinse (Figure 3).

The average UR pattern across feeds behaved similarly to a first-order kinetics model (Figure 2), with the differences (i.e., decrease in UR) between sequential rinses becoming smaller as the number of rinses increased. A similar pattern was also observed by Coblentz et al. [22] when evaluating the quantity of contaminants solubilized in the washing water of filter bags used for an in situ degradation assay. According to those authors, the main components of that contamination would include the particles of rumen digesta adhering to the bags and the ruminal microbes attached to feed particles.

The UR pattern obtained here disagrees with the statements of Vanzant et al. [16], who recommended a five-cycle (1 min each) rinsing procedure for bags used for in situ incubation in ruminants. However, that disagreement could be caused by differences between incubation environments. The bags used in situ are more susceptible to particle attachments caused by the direct contact with rumen contents, whereas rumen inoculum for in vitro procedures is filtered and also diluted in a clean buffer solution. Considering this, it seems logical that outside-bag contamination should be less for in vitro procedures, which would demand a lower number of rinses for cleaning when compared with in situ procedures.

Besides the UR decrease as the number of rinses increased, the variance among replicates also decreased (Figure 3), which brought evidence for the influence of contaminants on the random variation of the results, and that an adequate rinsing procedure can contribute to increasing experimental precision and repeatability. In general, the variance among replicates became stable and was minimized from the third rinse on, agreeing with the behavior of the UR across sequential rinses. This pattern brings into evidence another operational advantage of a machine-rising procedure. As it does not depend on hand operation, a standardized mechanical rinsing seems to act more homogenously on replicates and thus increases precision. It agrees with the statement by Paine et al. [23], who found smaller standard errors on average DM degradation when using a machine rinsing compared to a hand-rinsing procedure.

In summary, we concluded that a minimum of three 1 min cycles of machine rinsing are recommended for ruminal in vitro assays, which assures obtaining a stabilized apparently undigested residue with a minimized variance among replicates. This recommendation was added as a standard procedure in the method evaluated in the collaborative study.

### 3.2. Collaborative Study

**Figure 4:**
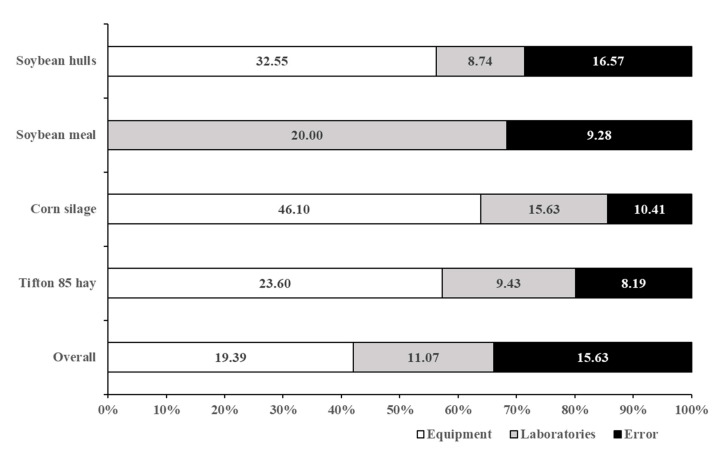
Comparative evaluation between variances of the in vitro dry matter digestibility associated with equipment type, laboratory, and error (between replicates) effects, according to different evaluated feeds (data label values are expressed as squared percentage units).

The total random variance of data was estimated as the sum of variances associated with equipment type, laboratories, and error (Figure 4). Even for the overall dataset, we did not include variance among feeds as a component of the total random variance. Variance between feeds is expected to occur, and it does not influence the performance of the method, as do equipment type or laboratory. On average, equipment type corresponded to 42% of the total random variance. A particular pattern was observed for soybean meal, where the model did not detect a positive variance between equipment type.

**Figure 5:**
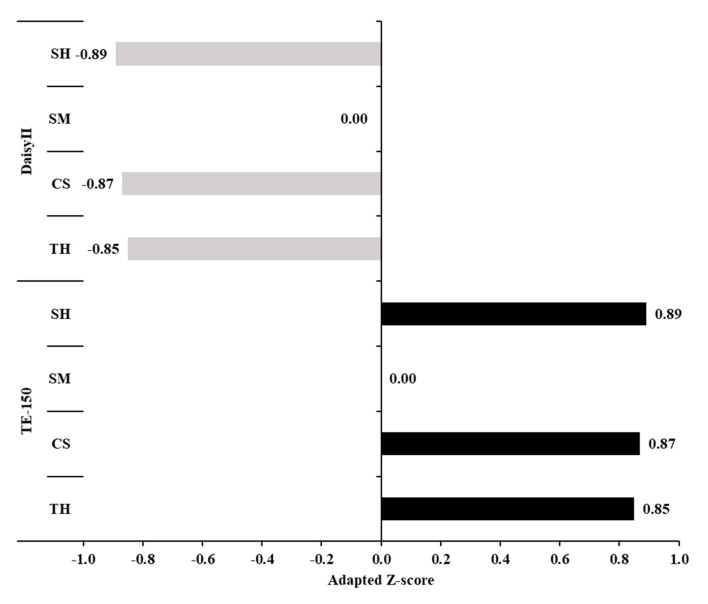
Adapted Z-scores for the in vitro dry matter digestibility for the evaluated equipment types with different feeds (TH, Tifton 85 hay; CS, corn silage; SM, soybean meal; and SH, soybean hulls). For details, see Equation (14).

This high contribution of equipment to the total random variance shows that equipment features can affect in vitro digestion estimates. On the other hand, this is a positive aspect in terms of method standardization and application, as this kind of influence can be anticipated and used to interpret and adjust the IVDMD estimates. Overall, the TE-150 had a positive effect, whereas DaisyII caused a negative effect on the IVDMD estimates (Figure 5). This pattern agreed with Silva et al. [2], who found greater IVDMD using TE-150 compared to DaisyII. Those fermenters presented some physical differences, including variations concerning jar rotation rate. This difference is critical, as it may affect the contact between filter bags and inoculum and, consequently, alter the IVDMD estimates. On average, the absolute difference between IVDMD obtained with DaisyII and TE-150 was 5.6 percentage points.

On average, the contribution of laboratory effect to the total random variance was 24% (Figure 4), being lower than the contribution of equipment (42%) and error (34%). This is the first evidence indicating that the method proposed here is reproducible and able to be adequately applied by different laboratories. It is important to notice that no laboratory behaved as an outlier (*p* > 0.05) according to the ranking performance test (Table 3), indicating aspects of robustness of the method, as laboratories did not exhibit a pronounced systematic error [20].

**Figure 6:**
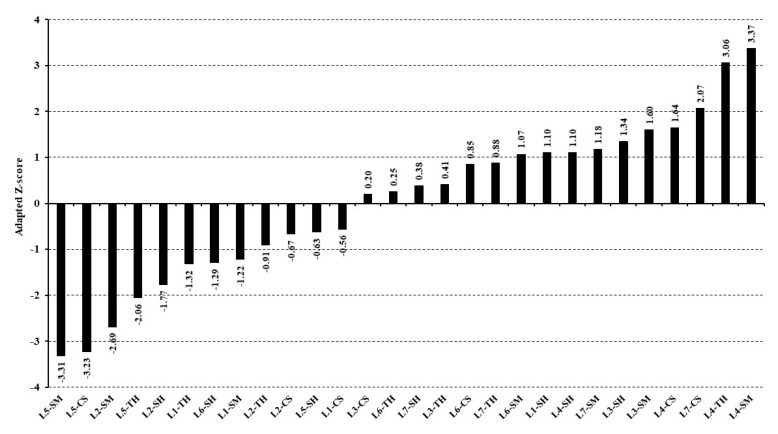
Adapted Z-scores for in vitro dry matter digestibility expressed according to different laboratories (L1–L7) and feeds (TH, Tifton 85 hay; CS, corn silage; SM, soybean meal; and SH, soybean hulls). For details, see Equation (14).

The individual performance of the laboratories was also evaluated, using the adapted Z-scores (Figure 6). Typically, the Z-scores are produced from the difference between each laboratory IVDMD and the overall mean of IVDMD divided by the standard deviation for each feed. However, in our study, there was a second factor contributing to differences among laboratories, which was the two types of artificial fermenters. Therefore, an adapted Z-score was calculated from eBLUPs, which were previously adjusted for the equipment type effect, allowing an unbiased comparison among laboratories regarding their performance.

As a general rule in a collaborative study, a satisfactory result is achieved when |Z| ≤ 2. Moreover, due to inherent and unavoidable variability among laboratories, a frequency of 80% of satisfactory results among laboratories is considered a successful performance [9]. However, the number of laboratories was limited in our study. Recommendations on the number of laboratories for a collaborative study range from a minimum of eight [24], to between eight and fifteen [25], to as many as possible [20]. Nonetheless, due to the characteristics of the proposed method, only seven laboratories made up the laboratory sample in our study.

Despite this, the Z-scores exhibited a sigmoidal pattern, which is an inherent characteristic of the normal distribution (Figure 6). Two of the Z-scores assumed marginal values very close to two (L5-TH and L7-CS). Assuming that those marginal values can be rounded down to two, then only five of the Z-scores showed unsatisfactory values. This means that approximately 82% of the Z-scores were found to be satisfactory, which provided further evidence of the adequate reproducibility of the evaluated method.

The repeatability ranged from 3.34 to 5.79% across feeds (Table 4) and fell within a range similar to that observed by other authors [2,10]. A common empirical approach in feed analysis laboratories is to consider that a replicate IVDMD analysis is acceptable if a maximum difference of 5% among duplicate aliquots is observed. Despite being a rule of thumb rather than a scientific approach, following this empirical reasoning leads to the conclusion that the observed repeatability for the proposed method is considered adequate in practical terms.

On the other hand, the reproducibility ranged from 5.93 to 8.94% across feeds (Table 4). At first glance, the observed reproducibility was very high when compared with the expected values of R predicted by the Horwitz equation [18].

In simple terms, the Re determined that the mean coefficient of variation among laboratories (i.e., reproducibility) increases by powers of two as the analyte level decreases by a power of 10. In other words, the Re doubles for every decrease of two orders of magnitude in the analyte concentration (expressed as a mass fraction). Such a pattern should be independent of either the nature of the analyte or the analytical technique that is used to make the measurement [17,18].

A direct evaluation of the observed R is obtained by calculating the HorRat, whose acceptable values must lie between 0.5 and 2.0 [17]. For the proposed method, HorRat ranged from 2.94 to 4.10 across feeds (Table 4). Generally, this would indicate that the proposed method is unacceptable concerning precision (i.e., reproducibility). However, it must be understood that the aforementioned limits for HorRat are not absolute, as transgressions are occasionally permitted in both directions [18].

**Figure 7:**
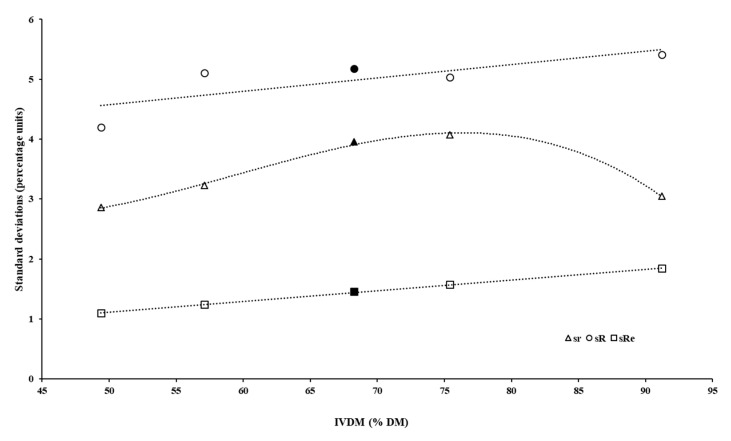
Descriptive pattern of the standard deviations of repeatability (sr), reproducibility (sR), and expected reproducibility (sRe) according to average values of in vitro dry matter digestibility (the central black data point corresponds to the mean digestibility for the overall dataset).

To understand the patterns of observed R and HorRat, a broader evaluation of the technical indicators of the method must be performed. Firstly, despite the fact that Re decreases as analyte concentration increases, the reproducibility expressed as absolute variation (i.e., as a standard deviation) must show a positive relationship with the concentration [17]. In fact, both sR and sRe showed a very similar pattern, according to IVDMD estimates (Figure 7), including very similar slopes (0.022 versus 0.018, respectively). Despite sR being, on average, 3.3 percentage units higher than sRe, their similar sensibility to analyte concentration variation indicates a functional agreement with the theoretical pattern of reproducibility parameters.

Secondly, r should ordinarily be approximately one-half to two-thirds of R [17]. This pattern was observed for the three feeds here evaluated, excepting soybean hulls (Table 4), which directly implied a high r/R for the overall dataset. At first glance, the r/R of 0.80 for soybean hulls could indicate that intra-laboratorial replications are so poor that they swamp the between-laboratory variation. However, a closer evaluation of the soybean hull IVDMD variability shows that the high r/R was not caused by a high sr (Table 4), and this pattern seems simply to reflect some particularity of this feed, which may affect that ratio without causing the variance of levels above that considered as normal and standard across feeds. Thus, despite the particular pattern of soybean hulls, the r-to-R ratios once more indicated that the proposed method has an adequate reproducibility.

However, the main aspect to be highlighted when interpreting both R and HorRat is the nature of the proposed method. The IVDMD is an analytical entity defined by the method itself (i.e., Type I method; [3]). A HorRat greater than two is commonly observed for this type of analytical entity, such as crude fat [26] and fibre [19]. This pattern is attributed to the fact that the Horwitz model does not apply to empirical analytes (i.e., those that are method-dependent), whose composition is ill-defined and whose concentration estimate depends on the specific details of the method [19]. In these cases, the fact that HorRat is >2.0 does not invalidate the method [26].

Due to differences in the cell wall digestibility and cell contents, the apparent undigested residue (Equation (4)) is mainly formed of fibrous compounds [27]. According to Horwitz et al. [19], fibre-related analytes are not chemically defined. In the presence of such an identity problem, the methods are necessarily empirical and accompanied by methodological and internal quality control problems that are reflected in high R values. Total gas production at fixed incubation times is strongly correlated with the extent of substrate digestion [28]. Some collaborative studies have found R values for gas production of 26.3% at 24 h, 15.4% at 48 h [29], and 8.2–9.4% at 72 h of incubation [30]. From this, the observed R values for IVDMD found in our study (3.34–5.79%, Table 4) can be considered low, and corroborate the reproducibility of the proposed method.

Moreover, the reproducibility limit represents the maximum acceptable difference between two single tests on identical test material with the same method in different laboratories with different operators using different equipment [31,32]. For all feeds, the maximum difference between laboratories did not exceed the reproducibility limit (Table 4), varying from 53 to 79%. This pattern adds to our previous arguments about the adequate reproducibility of the proposed method.

## 4. Conclusions

A standardized method for evaluating in vitro dry matter digestibility for ruminant feeds and diets was proposed and evaluated through a collaborative study with seven laboratories. The results highlighted that, if the method is followed exactly, its results are precise, and present adequate levels of repeatability and reproducibility.

## Figures and Tables

**Table 1:** Chemical composition of feeds used for evaluating in vitro dry matter digestibility.

Feed	Dry Matter ^1^	Crude Protein ^2^	Neutral Detergent Fibre ^2^
Tifton 85 hay	90.6	6.68	74.5
Corn silage	24.9	6.23	50.1
Soybean meal	88.4	47.9	24.0
Soybean hulls	88.0	15.4	66.4

^1^ % as fed. ^2^ % of dry matter.

**Table 2:** Average in vitro dry matter digestibility (%) of different feeds, according to the laboratories participating in the collaborative study.

	Feed ^1,2^			
Laboratory	Tifton 85 Hay	Corn Silage	Soybean Meal	Soybean Hulls
1	49.1 ± 0.70	58.0 ± 0.96	89.0 ± 0.89	79.6 ± 1.14
2	42.4 ± 0.85	48.5 ± 0.94	86.3 ± 0.61	65.3 ± 1.20
3	45.9 ± 0.57 ^3^	51.4 ± 0.76	94.2 ± 0.59	73.5 ± 0.87
4	56.2 ± 0.57	63.1 ± 0.46	97.4 ± 0.64	79.6 ± 0.61
5	47.8 ± 0.49	53.2 ± 0.54	85.1 ± 0.78	76.5 ± 0.91
6	51.6 ± 0.66	61.5 ± 0.67	93.2 ± 0.41	75.4 ± 0.74
7	52.6 ± 0.59	64.0 ± 0.65 ^4^	93.4 ± 0.76 ^3^	78.3 ± 0.76
Overall	49.4 ± 0.43	57.1 ± 0.56	91.2 ± 0.43	75.4 ± 0.52

^1^ Mean ± standard error. ^2^ Unless stated, within laboratories, the average values were calculated on *n* = 20. ^3^ *n* = 19. ^4^ *n* = 18.

**Table 3:** Ranking of the empirical best linear unbiased predictors for the effects of laboratories on in vitro dry matter digestibility of different feeds.

	Feed				
Laboratory	Tifton 85 Hay	Corn Silage	Soybean Meal	Soybean Hulls	Sum ^1^
1	6	5	5	2.5	18.5
2	5	6	6	7	24.0
3	3	4	2	1	10.0
4	1	2	1	2.5	6.5
5	7	7	7	5	26.0
6	4	3	4	6	17.0
7	2	1	3	4	10.0

^1^ Approximate two-tailed limits for the sum of ranking scores: 5, 27 (4 feeds, 7 laboratories, α = 0.05). For details, see Wernimont and Spendley [20].

**Table 4:** Estimates of variance components and technical indicators of the proposed method for in vitro dry matter digestibility, according to the evaluated feed.

		Feed			
	Overall	Tifton 85 Hay	Corn Silage	Soybean Meal	Soybean Hulls
Variance components [(%)^2^]					
Laboratories	11.07	9.43	15.63	20.00	8.74
Error	15.63	8.19	10.41	9.28	16.57
Technical indicators ^1^					
aIVDMD (%)	68.3	49.4	57.1	91.2	75.4
s_r_	3.95	2.86	3.23	3.04	4.07
r (%)	5.79	5.79	5.65	3.34	5.40
s_R_	5.17	4.20	5.10	5.41	5.03
R (%)	7.57	8.50	8.94	5.93	6.67
r/R	0.76	0.68	0.63	0.56	0.80
s_Re_	1.45	1.10	1.24	1.85	1.57
Re (%)	2.12	2.22	2.18	2.02	2.09
HorRat	3.57	3.83	4.10	2.94	3.19
RL	-	11.8	14.3	15.1	14.1
Δ_max_^2^	-	8.0 (68)	10.5 (73)	12.0 (79)	7.5 (53)

^1^ aIVDMD, average in vitro dry matter digestibility; s_r_, standard deviation within laboratories; r, repeatability; s_R_, standard deviation among laboratories; R, reproducibility; s_Re_, expected standard deviation of reproducibility; Re, expected reproducibility; HorRat, Horwitz ratio; RL, reproducibility limit (RL = 2.8 × sR); Δ_max_, maximum difference among the eBLUPs for IVDMD. ^2^ Values among parentheses expressed Δ_max_ as % of RL.

## Data Availability

The data generated during the current study are available from the corresponding author upon reasonable request.

## Supplementary Materials

The following supporting information can be downloaded at: https://www.mdpi.com/article/10.3390/ani12202842/s1, The method to evaluate in vitro dry matter digestibility is included as an Supplementary Material.
