# Supplementary material for: A Standard Procedure for In Vitro Digestion Using Rumen Fermenters: A Collaborative Study
Source: Animals (Basel). 2022 Oct 19;12(20):2842. doi: 10.3390/ani12202842 (PMC9597798; doi:10.3390/ani12202842)
Supplement: Supplementary file 1 [file animals-12-02842-s001.zip › animals-1959249-supplementary.pdf]

animals MDPI

Appendix

In vitro dry matter digestibility using artificial fermenters (models Daisy[II] Ankom, TE-150 Tecnal, or similar)

Apparatus

- Analytical balance (0.0001 g)

- Forced air-circulation oven

- Oven without forced-air circulation

- Non-woven textile (100 g/m2; 4 × 4.5 cm) or F57 (Ankom®) filter bags

- Sealer

- Desiccator

- Artificial fermenter

- CO_2 cylinder

- Cheesecloth

- Erlenmeyer flasks

- Volumetric flasks

- Blender

- Thermos flasks

- Digital Potentiometer

- Inoculum donor animal

- Washing machine

- Aluminum pan

- Aluminum trays

- Stove

Reagents

- Sodium bicarbonate ($NaHCO_3$) P.A.
- Monobasic sodium phosphate (Na_2HPO_4) P.A. anhydrous, dihydrate or heptahydrate
- Potassium chloride (KCl) P.A.
- Sodium chloride (NaCl) P.A.
- Magnesium sulphate ($MgSO_4$) heptahydrate P.A.
- Calcium chloride ($CaCl_2$) dihydrate P.A.
- Urea P.A.
- Neutral household detergent

Solutions

Buffer solution

- In a 1 L (10 L) volumetric flask containing 0.1 L (1 L) of distilled water, add 9.8 g (98 g) of $NaHCO_3$, 3.71 g (37.1 g) of anhydrous Na_2HPO_4 or 4 .65 g (46.5 g) of Na_2HPO_4 dihydrate or 7.00 g (70.0 g) of Na_2HPO_4 heptahydrate, 0.57 g (5.7 g) of KCl, 0.47 g (4.7 g) of NaCl, 0.12 g (1.2 g) of $MgSO_4$ heptahydrate, and 0.05 g (0.5 g) of $CaCl_2$ dihydrate. Shake for partial dissolution. Add 0.8 L (8 L) of distilled water and stir until completely dissolved. Make up the volume of the balloon to 1 L (10 L). This procedure should preferably be carried out 24 hours before incubation.

Urea solution

- Add 5.5 g of urea in a 100 mL volumetric flask. Add approximately 70 mL of distilled water and mix until completely dissolved. After the temperature has stabilized with the environment, complete the volume. The solution must be stored under refrigeration until utilization.

Neutral detergent solution

- In a 4 L Erlenmeyer, add 1 L of distilled water and 80 mL of neutral household detergent. Shake until completely homogenization and make up to volume with distilled water.

Procedures

In all procedures, use an analytical balance with an accuracy of 0.0001 g, on a special laboratory bench, and in a climate-controlled environment (20-25ºC or according to the manufacturer's specifications). Switch on the balance and wait 30 minutes for its stabilization.

Preparation of filter bags

1. Identify the filter bags with a permanent marker
2. Place the filter bags in an aluminium pan and add the neutral detergent solution in sufficient volume to cover all the bags
3. Take the aluminium pan on the stove and heat it up. The solution must be boiled for 15 minutes
4. Wash the filter bags under running water until the detergent is completely removed
5. Dry the filter bags in trays (thin layer without overlapping) sequentially in a forced air-circulation oven at 55°C for 24 hours and then in a non-ventilated oven at 105°C for 2 hours

6. Place the filter bags in a desiccator (maximum 20 units per procedure) and wait for the equilibrium to reach room temperature

7. Weigh the filter bags and record the weight. This will be the tare weight

8. Weigh approximately 500 mg of air-dried sample processed to pass through a 1-mm screen sieve, record the weight, and place inside the filter bag

9. Seal the filter bags using a sealer and set them aside for further incubation

10. For each incubation jar it is necessary to use at least two sealed blank filter bags (i.e., without sample)

Preparation of buffer solution

1. Add McDougall's buffer solution to an Erlenmeyer flask in sufficient volume to carry out the incubation procedure

2. Add urea solution to the McDougall's solution at a rate of 5 mL/300 mL. **This addition is considered to be optional, as the use of urea can decrease the discrimination power among samples (see details in Camacho et al., 2019). Therefore, whether or not to use urea is the only step here let to the analyst's discretion**

3. Attach a silicone hose to the CO_2 cylinder

4. Insert the free end of the hose into the buffer solution and release the CO_2 providing bubbling

5. Using the digital potentiometer, monitor the pH of the solution. The bubbling with CO_2 is ended when the pH is adjusted down to 6.8

6. Using an oven or climate-controlled room, acclimate the buffer solution to a temperature of 39°C

7. This procedure must be performed prior to the collection of rumen inoculum

Inoculum collection and processing

1. Turn on the fermenter heating (39°C) with empty jars

2. The inoculum can be collected from rumen canulated bovines; the production of inoculum from a pool of ruminal digesta obtained from three or more animals is recommended

3. The donor animal(s) must be adapted to the basal diet for at least 14 days before inoculum collection

4. The basal diet must consist of forage and concentrate in the proportion of 80:20 with a minimal of 12% of crude protein, both on dry matter basis. Mineral supplementation and water must be available *ad libitum.* The practice of fasting animals prior the collection should not be performed

5. Open the rumen cannula and collect portions of the solid and liquid parts of the digesta at different points on the rumen mat and store them in pre-heated thermos bottles (39°C)

6. Take the material immediately to the laboratory

7. Using a blender, mix the digesta for approximately 30 seconds

8. In an Erlenmeyer flask, filter the homogenized digesta through four layers of cheesecloth. Discard the material retained in the cheesecloth

9. Keep the filtrate (i.e., inoculum) acclimatized (39°C) using thermos bottles, oven, or climate-controlled room

10. This procedure must be performed immediately before starting the incubation

Incubation procedure

1. Remove the pre-heated jars from the fermenter and accommodate filter bags inside them. It is recommended that each jar operates with 20 to 25 filter bags containing samples and two extra blank filter bags

2. Add 400 mL of inoculum and 1600 mL of buffer solution to each jar

3. Quickly flush the headspace of each jar with CO_2 to replace atmospheric air with carbon dioxide. Cap the jars immediately

4. Place the jars in the fermenter, start the rotation mechanism and close the door. Make sure the heating is properly turned on and set at 39°C

5. Keep jars under standard temperature and rotation conditions for 48 hours. Check them regularly throughout the whole fermentation process

6. After 48 hours, open the fermenter and remove the filter bags

7. Wash them superficially with clean water and press them gently to remove gases

8. Place the filter bags in a washing machine, fill it with clean tap water, adjust it for delicate setting, and activate the washer for 1 minute. Drain water after washing. This procedure must be performed at least three times

9. After the washing cycles, gently press the filter bags to remove excess of water, which must be clear

10. Dry the filter bags in trays (thin layer without overlapping) sequentially in an oven forced air-circulation oven at 55°C for 24 hours and then in a non-ventilated oven at 105°C for 16 hours

11. Place the filter bags in a desiccator (maximum 20 units per procedure) and wait for equilibrium to reach room temperature

12. Weigh the filter bags and record the weight

Calculation of *in vitro* dry matter digestibility

Calculation of the residue obtained with blank filter bags

$$\mathrm{B} = \boldsymbol{F_B} - \boldsymbol{T}$$

where: B = contamination from the incubation procedure (g of dry matter); F_B = blank filter bag weight after incubation (g); and T = tare or pre-incubation filter bag weight (g).

The "blank" value to be used in the other calculations must be obtained by averaging the contamination (B) obtained in the blank filter bags evaluated within the incubation run. It is important to emphasize that contamination is inherent to each incubation procedure. Therefore, blanks are required for each procedure, and it is not possible to use "average values" obtained from previous procedures.

Calculation of apparently undigested residue:

$$\mathbf{U} = \boldsymbol{F_R} - \boldsymbol{T}$$

where U is the apparently undigested residue (g of dry matter), F_R is the weight of the filter bag containing the residue after incubation (g), and T is tare or pre-incubation filter bag weight (g).

Calculation of *in vitro* dry matter digestibility:

$$\%\boldsymbol{U_{ADS}} = \left(\frac{\boldsymbol{U} - \boldsymbol{B}}{\boldsymbol{ADS}}\right) \times \mathbf{100}$$

$$\%\boldsymbol{U_{DM}} = \frac{\%\boldsymbol{U_{ADS}}}{\%\boldsymbol{DM_{105}}} \times \mathbf{100}$$

$$\%\boldsymbol{IVDMD} = \mathbf{100} - \%\boldsymbol{U_{DM}}$$

where $\%U_{ADS}$ is the apparent undigestibility based on the air-dried sample (%), $\%U_{DM}$ is the apparent undigestibility based on dry matter (%), ADS is the air-dried sample mass (g), %IVDMD is the *in vitro* dry matter digestibility (%), and $\%DM_{105}$ is the percentage of dry matter of the sample evaluated at 105°C. The other terms were previously defined.

When displaying the results, it is mandatory to report the following information: 1. brand and model of the artificial fermenter, 2. type of filter bag used, and 3. addition or not of urea to the buffer solution. These three factors will affect digestibility estimates and their knowledge is crucial for interpretation and comparative evaluation of results from different assays.
